# Novel Genetic Melanoma Vaccines Based on Induced Pluripotent Stem Cells or Melanosphere-Derived Stem-Like Cells Display High Efficacy in a murine Tumor Rejection Model

**DOI:** 10.3390/vaccines8020147

**Published:** 2020-03-26

**Authors:** Agnieszka Gąbka-Buszek, Eliza Kwiatkowska-Borowczyk, Jakub Jankowski, Anna Karolina Kozłowska, Andrzej Mackiewicz

**Affiliations:** 1 Chair of Medical Biotechnology, Poznan University of Medical Sciences, 8, Rokietnicka Street, 60-806 Poznan, Poland; elizakwi@gmail.com (E.K.-B.); jankowskij7@gmail.com (J.J.); annakozlowsk@gmail.com (A.K.K.); andrzej.mackiewicz@wco.pl (A.M.); 2 Department of Diagnostics and Cancer Immunology, Greater Poland Cancer Centre, 15, Garbary Street, 61-866 Poznan, Poland

**Keywords:** cancer immunotherapy, therapeutic vaccine, melanoma, cancer stem cells, induced pluripotent stem cells

## Abstract

Therapeutic cancer vaccines have elicited renewed interest due to the development of immune checkpoint inhibitors. The role of these vaccines is to induce specific effector cells for killing cancer cells. Cancer stem cells (CSCs) are responsible for tumor growth and progression. Accordingly, they are targets for various cancer therapies, including immunotherapy. Here, we demonstrate the effectiveness of melanoma vaccines composed of genetically modified tumor cells admixed with melanoma stem-like cells (MSC) or induced pluripotent stem cells (iPSCs). Two vaccines were constructed. The first vaccine contained cells derived from B16F10 melanospheres (SFs) with CSC characteristics. The second vaccine contained syngeneic murine induced pluripotent stem cells (miPSCs). iPSCs or SF cells were admixed with B16F10 cells, modified with the designer cytokine Hyper-IL6(H6) (B16/H6). Control mice received B16/H6 cells, B16F10 cells or PBS. Immunization with either vaccine significantly inhibited tumor growth and increased disease-free survival (DFS) and overall survival (OS) in C57BL/6 mice. Mice treated with the SF or iPSC vaccine demonstrated increased activation of the immune response in the vaccination site and tumor microenvironment compared to those treated with B16/H6, B16F10 or PBS. Higher infiltration of dendritic cells (DCs) monocytes, and natural killer (NK) cells; lower numbers of myeloid-derived suppressor cells (MDSCs) and regulatory T cells (Tregs); higher levels of the cytokines INFγ and IL-12 were observed with the novel vaccines than with the control treatments. In vitro restimulation of splenocytes derived from mice immunized with B16F10 cell, SF cell or miPSC lysates increased the proliferation of CD4+ T helper lymphocytes and secretion of cytokines. An increased serum titer of antibodies directed against B16F10 cells was found in mice immunized with the SF vaccine. The most effective DFS and OS extensions were reached with the miPSCs vaccine. The described results form the basis for a novel platform for the next generation of cancer vaccines composed of allogeneic cancer-specific cells modified with a molecular adjuvant gene and admixed with allogeneic miPSCs or SFs.

## 1. Introduction

The advanced metastatic melanoma is still a deadly disease. Recent developments in targeted and immunetherapies, such as immune checkpoint inhibitors, led to the statistically significant extension of overall survival (OS) of a fraction of metastatic melanoma patients. However, the clinical benefits were often temporary and relatedwith serious adverse events [1].

Cancer-initiating or cancer stem cells (CSC) area small fraction of cells which play an important role in cancer progression [2]. CSCs are characterized by a low degree of differentiation, capacity for self-renewal, potential for the rapid restoration of tumor cells pool and the expression of antigens other than in differentiated tumor cells, but similar to those in normal stem cells. CSCs are involved in tumor recurrence and metastasis, and are resistant to conventional therapies, such as chemo- or radiotherapy. Accordingly, they are the targets for novel therapeutic strategies, including immunotherapies [3].

We previously developed a human, allogeneic, genetically modified, whole-cell melanoma vaccine—the only one in its class (AGI-101H) [4], and this vaccine, in clinical trials in advanced-stage (stage IIIB-IV) resected [5] and non-resected melanoma [6], demonstrated a high response rate, extended disease-free survival (DFS) and long-term OS in treated patients.

AGI-101H comprises two human melanoma cell lines (Mich1 and Mich2), genetically modified with the designer cytokine Hyper-IL6 (H6). H6 cDNA was stably transduced into both cell lines using retrovirus [7]. H6 is a fusion protein comprising Interleukin 6 (IL-6) linked with its soluble receptor α (sIL-6R; also known as gp80 and CD126), which is able to directly target gp130 (CD130), the signal-transducing β subunit of the IL-6-like cytokine receptor complex [8]. H6 displays a very broad spectrum of biological activities, since it activates the JAK1/STAT3, MPK and PI3K pathways in a trans-signaling manner [9]. Accordingly, the activity of H6 is significantly broader than that of IL-6 alone, since the IL-6 binding receptor subunit (gp80) is only expressed on hepatocytes and the site of vaccine administration. It enhances the host T cell allogeneic response and activates some specific leukocyte subtypes [9]. H6 serves as a molecular adjuvant for AGI-101H and is secreted at the CD8+ and CD4+ T cell responses, blocks FoxP3 expression in CD25+CD4+ cells, inhibits the formation of regulatory T cells (Tregs), and induces dendritic cells (DC) maturation and cryptic antigen presentation [9], as well as the production of GM-CSF [10]. Downstream, H6 affects the activation of memory CD8+ and CD4+ T cells and natural killer (NK) cells and the inhibition of myeloid-derived suppressor cells (MDSCs) (data not published) [11]. Moreover, due to the expression of the gp130 receptor on the cells composing the vaccine, chronic autocrine exposure to H6 leads to activation of the JAK1/STAT3-P/Oct4 pathway, which changes the phenotype of these cells towards a melanoma stem cell-like phenotype [11,12]. These cells acquire high aldehyde dehydrogenase (ALDH) activity, increased expression of CD44 and the decreased expression of tyrosinase and microphthalmia-associated transcription factor (MITF) [11]. Recently, we demonstrated that AGI-101H induces ALDH1A1-specific, functional cytotoxic T cells, and stimulates anti-ALDH1 antibody production, which strongly suggests its immune-mediated targeting of melanoma stem-like cells (MSCs) [11]. Accordingly, the overall goal of the current study was to verify the hypothesis that enrichment of a whole-cell melanoma vaccine with stem cells increases the potential of the vaccine to generate a specific antimelanoma immune response. For this purpose, we adopted a murine tumor rejection model, in which we tested a murine melanoma vaccine mimicking the human vaccine (AGI-101H), containing stem cells. We constructed two vaccine variants. For the first variant, as the source of MSCs, we generated melanospheres, using B16F10 cell culture under nonadherent conditions and medium for suspension culture [13,14]. Cells acquired from the melanospheres displayed a phenotype similar to that of MSCs, as revealed by the decreased expression of MITF and Tyrosinase, the increased expression of vascular endothelial growth factor (VEGF), and high ALDH activity, compared with parental B16F10 cells. The second vaccine contained commercially available induced pluripotent stem cells (miPSCs), derived from C57BL/6 mice. These cells were subjected to magnetic sorting for the SSEA-1 expression, due to the low activity of alkaline phosphatase and the low expression of SSEA-1. B16F10 melanoma cells, which are syngeneic to C57BL/6 mice, were modified with H6 cDNA and admixed with cells derived from B16F10 melanospheres (SFs) or miPSCs, derived from the same mouse strain at a 1:2 ratio.

In both experimental settings, immunization with the vaccines containing stem cells led to significant reductions in tumor growth and increases in DFS and OS in treated mice. The most effective DFS and OS extensions were achieved with the vaccine containing miPSCs. The last finding, beyond validating the mode of action of AGI-101H, forms the basis for a novel approach for the next generation of cancer vaccines composed of allogenic cancer-specific cells (such as cells from melanoma tumors or other tumors), modified with a molecular adjuvant gene and admixed with allogeneic miPSCs.

## 2. Materials and Methods

### 2.1. Mice

Five- to six-week-old C57BL/6 male mice were purchased from Charles River Laboratories (Sulzfeld, Germany). Mice were maintained under constant pathogen-free conditions and a 12-hour day/night cycle, with unrestricted access to food and water. Experiments were initiated after a two-week quarantine. All experiments were carried out at least twice in accordance with the national and institutional guidelines for humane treatment of laboratory animals, after obtaining the consent of the Local Ethics Committee for Experiments on Animals in Poznań (No. 67/2012).

### 2.2. Cell lines and Culture

Murine B16F10 melanoma cells were purchased from ATCC (ATCC, Manassas, VA, USA). Cells were grown in monolayer culture in high-glucose DMEM with stable glutamine (Sigma Aldrich, St. Louis, MO, USA), supplemented with 10% fetal bovine serum (FBS), and 1x Antibiotic/Antimycotic Solution (Sigma Aldrich, St. Louis, MO, USA). Mouse embryonic fibroblasts (MEFs) were purchased from Tebu Bio (Berlin, Germany) and cultured in high-glucose DMEM with stable glutamine (Sigma Aldrich, St. Louis, MO, USA), supplemented with 15% FBS and 1x Antibiotic/Antimycotic Solution (Sigma Aldrich, St. Louis, MO, USA). MEFs were grown to the appropriate number, harvested, irradiated with a dose of 80 Gy (Gamma-Cell 1000, RTA, Best Theratronics Ottawa, Canada), aliquoted and cryopreserved (50% FBS, 40% DMEM, and 10% DMSO). miPSCs were purchased from System Bioscience, sorted with an anti-SSEA-1 antibody using magnetic bead sorting (Miltenyi, Bergisch Gladbach, Germany) and grown on an inactivated MEF layer in KnockOut DMEM (Gibco), supplemented with 15% KnockOut Serum Replacement KSR, (Thermo Fisher Scientific, Waltham, MA, USA), 2 mM L-glutamine (Thermo Fisher Scientific, Waltham, MA, USA), 1% Antibiotic/Antimycotic Solution (Sigma Aldrich, St. Louis, MO, USA), 1% Non-Essential Amino Acids (NEAA); (Merck Millipore Burlington, MA, USA) and 1000 U/mL leukemia inhibitory factor (LIF) (Merck Millipore Burlington, MA, USA). MiPSCs were passaged every 2–3 days using 1 x TrypLE Select Enzyme (Thermo Fisher Scientific, Waltham, MA, USA). MEFs were removed by 20 minutes of preincubation of the cell suspension on a 0.1% gelatin-coated plate. After 20 minutes, the nonadherent cell fraction (miPSCs) was collected and plated on fresh feeder cells at a density of 18 × 10^3^/cm^2^.

MiPSCs were cultured to the appropriate confluency under conditions to maintain their pluripotent state, irradiated with an 80-Gy dose, aliquoted and cryopreserved (50% FBS, 40% DMEM, and 10% DMSO).

### 2.3. Transduction of B16F10 Cells with H6 cDNA

An adenoviral system, in contrast to the retroviral vector used in our human AGI-101H vaccine, was used to limit the exposure of B16F10 cells to H6, to prevent their conversion towards an MSC-like phenotype. In these studies, H6 was used only as a molecular adjuvant. An E1-deleted recombinant adenovirus of human strain 5 encoding H6 (AdH6) was constructed at the Department of Cancer Immunology at the University of Medical Sciences (Poznan, Poland). The virus was propagated and titrated on E1-transfected 293 cells, as described previously [15]. B16F10 cells at 90% confluence were transduced with AdH6 for 24 h. Then, the cells were harvested, irradiated with a dose of 80 Gy, aliquoted and stored in liquid nitrogen.

### 2.4. Melanosphere Culture

B16F10 cells were cultured under conditions that induce melanosphere formation. The cells were cultured in ultra-low attachment flasks (Corning, New York, USA) in medium consisting of DMEM/F12 (Thermo Fisher Scientific, Waltham, MA, USA), supplemented with GlutaMAX, 2% B27 supplement (Thermo Fisher Scientific, Waltham, MA, USA)), and 1x Antibiotic/Antimycotic Solution. Immediately before use, recombinant basic mouse Fibroblast Growth Factor (bFGF) ((Thermo Fisher Scientific, Waltham, MA, USA)) and recombinant mouse Epidermal Growth Factor (EGF) (Thermo Fisher Scientific, Waltham, MA, USA) were added to final concentrations of 20 ng/mL and 10 ng/mL, respectively [13,14]. Melanospheres were cultured for 10 weeks and passaged weekly. The expression of MITF, Tyrosinase, NANOG, VEGF, and the phosphorylated signal transducer and activator of transcription 3 (STAT3) and ALDH activity were analyzed.

### 2.5. Phenotypic Analysis of miPSCs

The Human and Mouse Pluripotent Stem Cell Analysis Kit (BD Biosciences, San Jose, CA, USA) was used for cytometric analysis (FACSCanto flow cytometer, BD Biosciences, San Jose, CA, USA). Immunocytochemical staining was also performed to verify the parental cell phenotype using anti-SSEA-1 (Merck Millipore Burlington, MA, USA), anti-Epcam (Abcam, Cambridge, UK), anti-E-cadherin (Abcam, Cambridge, UK), and anti-NANOG (Abcam, Cambridge, UK) antibodies at a dilution of 1:250 in 0.1% normal goat serum(NGS)(Thermo Fisher Scientific, Waltham, MA, USA) in PBS.

### 2.6. MITF and Tyrosinase Cytometric Assays

MITF and Tyrosinase expression levels were determined by flow cytometry. Staining was performed with the BD Transcription Factor Buffer Set, according to the manufacturer’s protocol. The following antibodies were used: PE-conjugated goat anti-mouse Tyrosinase, PE-conjugated goat anti-mouse IgG isotype control, mouse anti-MITF, FITC-conjugated rat anti-mouse IgG1κ, and FITC-conjugated mouse IgG1κ isotype control (BD Biosciences, San Jose, CA, USA). Analyses were carried out using a flow cytometer (FACSCanto, BD Biosciences, San Jose, CA, USA).

### 2.7. Surface Antigen Staining

In total, 100,000 cells were washed with PBS and resuspended in a PBS-antibody solution. After 30 minutes of incubation at 4 °C, the cells were washed again and analyzed with a flow cytometer. The following antibodies were used (BD Biosciences, San Jose, CA, USA): PE-conjugated rat anti-CD274, PE-conjugated rat anti-I-A/I-E, PE-conjugated rat IgG2b κ isotype control, and PE-conjugated rat IgG2a κ isotype control.

### 2.8. Real-Time PCR

Total RNA from the cells was isolated with TriReagent (Sigma Aldrich, St. Louis, MO, USA) according to the manufacturer’s protocol. cDNA was syntetized from 1 µg RNA with iScript™ cDNA Synthesis Kit (BioRad, Hercules, CA, USA). Briefly, a mixture containing 4 μL of iScript Reaction Mix, 1 μL of reverse transcriptase (BioRad, Hercules, CA, USA), 1 μg of RNA and water, to a final volume of 15 μL, was prepared for reverse transcription. The mixture was incubated at 25 °C for 5 minutes, 42 °C for 30 min and 85 °C for 5 min.

Probes and primers were designed using the Universal Probes Library (Roche, Basel, Switzerland). Probes were purchased from Roche, and primers were purchased from Sigma Aldrich.

The following primers and probes (in brackets) were used: Gene: Oct 3/4 (F: GAGGCTACAGGGACACCTTTC; R:GTGCCAAAGTGGGGACCT) (6), SSEA-1 (F: TGGTACTACGCGTGTTCGAC; R: CCAGGGCTTTGCCAGTTA) (32), NANOG (F:GCCTCCAGCAGATGCAAG; R: GGTTTTGAAACCAGGTCTTAACC) (25), and VEGFa (F: AAAAACGAAAGCGCAAGAAA; R: GGAACAAGTCTCGCCTCTTT) (1). Real-time PCR was performed using a Probes Master kit (Roche, Basel, Switzerland) on the LightCycler 480 (Roche, Basel, Switzerland).

The following cycle was used: initial denaturation at 95 °C for 10 minutes, denaturation at 72 °C for 10 seconds, annealing at 58 °C for 30 seconds, extension at 72 °C for 1 second; 45 cycles (denaturation, annealing, and extension).

The results were normalized to the Gapdh expression level and are presented as the fold change relative to the expression in an adherent culture of B16F10 cells (for melanospheres) or embryonic stem cells (ESCs) (for miPSCs).

### 2.9. Determination of Aldehyde Dehydrogenase Activity

ALDH activity was investigated using the ALDEFLUOR™ Kit (STEMCELL Technologies Vancouver, Canada), according to the manufacturer’s instructions. Briefly, 200,000 cells were washed with PBS and suspended in 1 mL of assay buffer. Five microliters of activated ALDEFLUOR reagent was added to the cells, and half of the total volume was transferred to a tube containing DEAB reagent (negative control). The samples were incubated at 37 °C for 30 min, centrifuged, resuspended in the assay buffer and analyzed with a flow cytometer.

### 2.10. Alkaline Phosphatase Activity Detection

The Alkaline Phosphatase Detection Kit (Merck Millipore Burlington, MA, USA) was used to evaluate enzymatic activity. The reaction mixture consisted of naphthol phosphate, fast red violet and water in a 2:1:1 ratio. After 15 min of incubation, the number of positive colonies was analyzed.

### 2.11. Western Blot Analysis

Proteins were isolated from cell lysates generated by cell lysis with RIPA buffer and centrifugation. In total, 30 μg of protein wasrun on a 5% polyacrylamide gel at 200 V, transferred to a PVDF membrane and blocked in 5% powdered milk. The membranes were incubated overnight with primary antibodies against STAT3 (1:2000, Cell Signaling Technology, Danvers, MA, USA) and phospho-STAT3 (1:2000, Cell Signaling Technology, Danvers, MA, USA). After washing, the membranes were incubated for an hour with secondary antibodies (1:2000, Santa Cruz Biotechnology, Dallas, TX, USA) or an anti β-actin antibody (1:1000, Santa Cruz Biotechnology, Dallas, TX, USA).

### 2.12. Vaccine Composition

Three therapeutic vaccines and two controls were constructed. The SF/H6 vaccine consisted of 1 × 10^6^ cells obtained from SFs, admixed with 5 × 10^5^ B16H6 cells (B16F10 cells expressing H6). The miPSC/H6 vaccine consisted of 1 × 10^6^ miPSCs, admixed with 5 × 10^5^ B16H6 cells. B16/H6, which consisted of 1 × 10^6^ wild-type B16F10 cells admixed with 5x × 10^5^ B16H6 cells; B16, which was a control that consisted of 1.5 × 10^6^ wild-type B16F10 cells; and PBS, which was a control that consisted of 200 μL of PBS buffer, were also used. All cells were irradiated with a dose of 80 Gy.

### 2.13. Immune Response at the Site of Vaccine Administration

Mice (6 animals per group) were immunized subcutaneously (s.c.) twice, with an intervening period of two weeks (Figure 2A). The cells in the first dose were suspended in PBS (200 μL), while those in the second dose were suspended in Matrigel (200 μL) (BD-Matrigel^®^ Matrix Basement Membrane High Concentration). The Matrigel plugs were resected 4 days later (Figure 2A), pooled for each research subgroup, cut into pieces and incubated at 37 °C for 30 min, with a mixture of collagenase (1 mg/mL, Roche, Basel, Switzerland), DNase (0.3 mg/mL, Roche, Basel, Switzerland) and dispase (50 U/mL, Corning, New York, USA). The digested material was cooled on ice, passed through a Falcon^®^ 70-µm cell strainer (BD) and centrifuged. The supernatants were collected and stored at −80 °C for further cytokine analysis. The cells were then washed in PBS, counted, stained with monoclonal antibodies and analyzed by flow cytometry. The following monoclonal antibodies (mAbs) were used: APC-conjugated rat anti-mouse F4/80 (BioLegend, San Diego, CA, USA), PECy7-conjugated rat anti-mouse Ly6C (BioLegend, San Diego, CA, USA), PE-conjugated rat anti-mouse CD11b (BD), PerCP/Cy5.5-conjugated rat anti-mouse CD45 (BioLegend, San Diego, CA, USA), PE-conjugated rat anti-mouse CD49b (BD).The concentrations of proinflammatory cytokines (IL-12p70 and INFγ) were analyzed using a Cytometric Bead Array (CBA) mouse inflammation kit (BD Biosciences, San Jose, CA, USA) according to the manufacturer’s protocol.

### 2.14. Analysis of Immune Cells Isolated from Spleens

Ten days after the second dose administration, spleens from each study subgroup (5 animals per group) were collected, pooled (Figure 3A), minced, and filtered through a 70-μM cell strainer (BD Biosciences, San Jose, CA, USA). The erythrocytes were lysed using ACK Lysis buffer. The relative amounts (%) of CD4+ T cells, CD8+ T cells, memory cells, MDSCs and CD4+ Foxp3+ Tregs in the pooled spleen samples, were assessed and compared. The following mAbs were used: FITC-conjugated hamster anti-mouse CD3 (BD Biosciences, San Jose, CA, USA), PECy7-conjugated rat anti-mouse CD4 (BD), APC-conjugated hamster anti-mouse CD62L (BioLegend, San Diego, CA, USA), PE-conjugated rat anti-mouse CD44 (BD), PE-conjugated hamster anti-mouse CD3 (BD Biosciences, San Jose, CA, USA), FITC-conjugated rat anti-mouse CD4 (BD Biosciences, San Jose, CA, USA), PECy7-conjugated rat anti-mouse CD8 (BioLegend, San Diego, CA, USA), APC-conjugated rat anti-mouse CD25 (BioLegend, San Diego, CA, USA), APC-conjugated hamster anti-mouse CD3 (BioLegend, San Diego, CA, USA), PE-conjugated rat anti-mouse CD62L (BioLegend, San Diego, CA, USA), and PerCP/Cy5.5-conjugated hamster anti-mouse CD69 (BD Biosciences, San Jose, CA, USA).

### 2.15. T Helper Cell Cytokine Release and Proliferation Assays

Cells isolated from spleens were seeded in triplicate in 96-well plates at a concentration of 1 × 10^6^ cells per well and restimulated with lysates of B16, SF (SF/H6 study group) cells or miPSCs (miPSC/H6 study group), at a ratio of 2:1. Lysates were prepared by freezing and thawing the cells suspended in X-vivo medium five times. The suspension was centrifuged and the supernatant was frozen at −80 °C. Positive controls were stimulated with anti-CD3/CD28 antibodies, while negative controls were not stimulated. After 72 h, 50 μL of medium was harvested for cytokine analysis, and 50 μL of x-vivo medium containing 3H-thymidine was added at 1 μC per well. After approximately 18 h, the cells were harvested (PerkinElmer), and lymphocyte proliferation was measured using a β scintillation counter (1450 Luminescence Counter Micro Beta TriLux, PerkinElmer, Waltham, MA). The concentrations of cytokines were determined using the Mouse Th1/Th2/Th17 Cytokine CBA Kit (BD Biosciences, San Jose, CA, USA).

### 2.16. Tumor Cell Binding by Immune Serum

Blood was collected 10 days after the second immunization by retro-orbital puncture, pooled in each study subgroup (5 animals per group), and the serum was prepared. IgG levels in serum samples from vaccinated and control mice were assessed using the Mouse IgG total ELISA Ready-SET-Go! Kit (eBioscience, San Diego, CA, USA). Analyses were performed according to the manufacturer’s instructions. Microplates were read at 450 nm with a 96-well ELISA plate reader (ELX808, Bio-Tek Instruments Winooski, VT, USA). B16F10 cells were washed with PBS+0.1% sodium azide, blocked with anti-CD16/CD32 antibodies (BD Biosciences, San Jose, CA, USA), and incubated with plasma, with an equal quantity of IgG for 60 minutes on ice. The cells were washed again and incubated with PE-conjugated anti-mouse IgG (BioLegend, San Diego, CA, USA), for 30 minutes at 4°C. After washing, the cells were analyzed using a FACSCanto flow cytometer (BD Biosciences, San Jose, CA, USA) for the binding of serum IgG.

### 2.17. Immune Response in the Tumor Microenvironment

To analyze cells infiltrating tumors, mice were immunized twice with an intervening period of 14 days (Figure 6A). Seven days after the second immunization, the mice were challenged s.c. with 1 × 10^5^ B16F10 cells suspended in 200 µL of Matrigel. Seven days after B16F10 cell injection, the tumors in the Matrigel were excised and pooled for each research subgroup (5 animals per group) (Figure 6A,B). Infiltrating cells were recovered by enzymatic digestion with collagenase (1 mg/mL, Roche, Basel, Switzerland), DNase (0.3 mg/ml, Roche, Basel, Switzerland) and dispase (50 U/mL, Corning, New York, USA), as described previously. The cells were then washed in PBS, counted, and stained with monoclonal antibodies. The following antibodies were used: PerCP/Cy5.5-conjugated rat anti-mouse CD45 (BioLegend, San Diego, CA, USA), APC-conjugated rat anti-mouse F4/80 (BioLegend, San Diego, CA, USA), PECy7-conjugated rat anti-mouse Ly6C (BioLegend, San Diego, CA, USA), PE-conjugated rat anti-mouse CD11b (BD), APC-conjugated hamster anti-mouse CD11c (BioLegend, San Diego, CA, USA), PE-conjugated rat anti-mouse CD49b (BD), FITC-conjugated rat anti-mouse CD4 (BD), and PE-conjugated human anti-mouse Foxp3 (BioLegend, San Diego, CA, USA). The cells were analyzed with flow cytometry using a FACS Canto (BD Biosciences, San Jose, CA, USA). Proinflammatorycytokine concentrations were analyzed using a CBA mouse inflammation kit (BD Biosciences, San Jose, CA, USA), according to the manufacturer’s protocol.

### 2.18. Tumor Growth, Disease-Free survival and Overall Survival in Immunized Mice.

Mice were immunized twice, with an intervening period of 14 days (Figure 7A). Seven days after the second immunization, the mice were challenged s.c. with 1 × 10^5^ B16F10 cells, suspended in 100 µL of HBSS buffer (Sigma Aldrich, St. Louis, MO, USA). Three and seven days later, the mice were again immunized (Figure 7A). After the appearance of tumors, they were measured every 2–3 days. Tumor volume was calculated from the formula L * W * H * π/6, where L means length, W width, H height. Mice were euthanized when tumors reached a volume of about 2000 mm^3^, or because of visible pain or illness.

### 2.19. Statistical Analyses

Data were analyzed with Prism 6.01 statistical software ((GraphPad Software, San Diego, CA, USA). Experiments were performed at least twice. Data were analyzed by Student’s t-test when comparing two groups. For data with a non-Gaussian distribution, a Mann-–Whitney test was performed to determine statistical significance. Comparisons of means among groups of mice were made by one- or two-way ANOVA and the Tukey post-hoc test. Survival curves were analyzed using the log-rank test (Mantel-Cox). Experiments with animals were performed at least twice, using a minimum of five mice per group. *P* values < 0.05 were considered statistically significant.

## 3. Results

### 3.1. Melanosphere Cells Demonstrate a Melanoma Stem Cell-Like Phenotype

**Figure 1:**
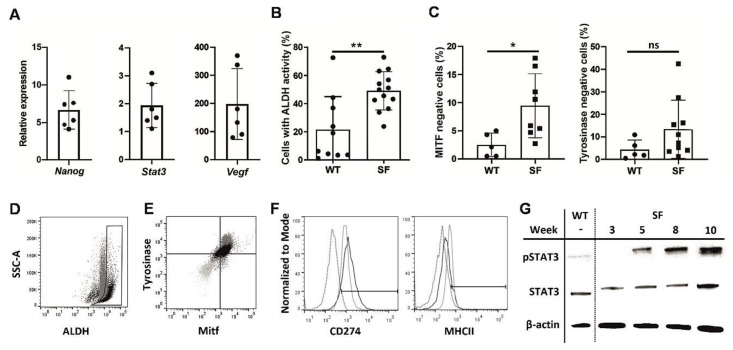
Phenotypic analysis of melanoma cells cultured as melanospheres SFs.The B16F10 melanoma cell line was cultured for 10 weeks under nonadherent conditions in enriched medium, as described in the Methods section, and analyzed weekly to assess the expression of cancer stem cell markers. Quantitative PCR was used to assess Nanog, Stat3, and Vegf expression in the SFs during weeks 5–10 of culture. The results were normalized to the Gapdh expression level and are presented as the fold change, relative to the expression in an adherent culture of B16F10 cells (WT) (**A**). Cytometric assay of the number of cells with highly active aldehyde dehydrogenase ALDH in SF and wild type WT cultures, passages 6-10 from three culture cycles; (** *p* < 0.01) (**B**). Percentages of microphthalmia-associated transcription factor MITF- and tyrosinase-negative cells in SF and WT cultures measured by flow cytometry; (* *p* < 0.05) (**C**). Representative ALDH activity graph, comparing SF (black) and WT (gray) cultures. Gate drawn according to WT cells with inactivated ALDH (**D**). Representative MITF- and tyrosinase expression graph, comparing SF (black), WT (gray) and isotype control (light gray) cultures (**E**). Expression of CD274 and MHCII in SF (black) and WT (gray) cultures, compared to isotype control cultures (dotted) (**F**). Western blot results for Y705 phosphorylated and total STAT3 protein were normalized to the housekeeping gene β-actin in WT and SF cultures at various time points (**G**).

SFs displayed a number of CSC characteristics compared to adherent B16F10 cultures, a tendency that strengthened with consecutive passages. Nanog mRNA expression [16], which often occurs in melanocytes early in development, as well as the expression of Stat3 and VEGF, which are often associated with cancer survivability [17], were increased in late melanosphere passages compared to similar passages of WT cells (Figure 1A). Similarly, high ALDH activity, which is common in CSCs [18], was observed in SFs (Figure 1B,D), while the expression of differentiated melanocyte markers, such as MITF and tyrosinase, was downregulated in the melanospheres (Figure 1C,E). The MHC II level was decreased and CD274 level was increased in SFs, suggesting an enhanced potential to attenuate the immune response, which is another CSC hallmark [19] (Figure 1F). Finally, STAT3 phosphorylation was more pronounced in SFs than in WT cells and increased with time (Figure 1G). Combined, these findings clearly show the phenotypic similarities between SFs and CSCs.

### 3.2. MiPSC Characteristics

MiPSCs cultured under conditions supporting pluripotency expressed SSEA-1, Epcam, E-cadherin, NANOG and alkaline phosphatase, which are considered early markers of pluripotency (Figure S1). Real-time PCR demonstrated a higher or equal expression of Oct 3/4 and Ssea-1 in miPSCs than in embryonic stem cells (Figure S1).

### 3.3. Cellular Infiltrates and Cytokines in the Vaccination Site and Spleens

**Figure 2:**
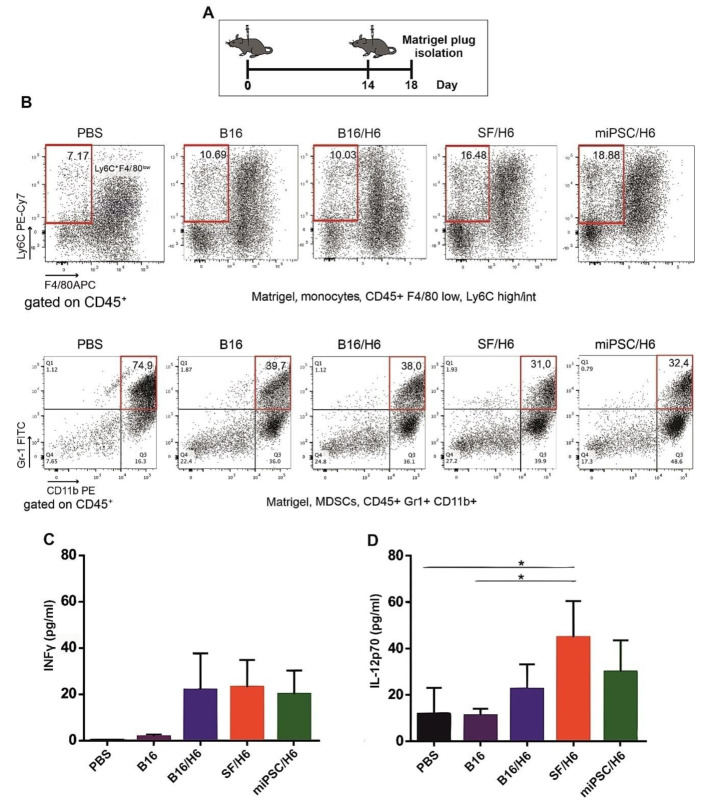
Mice were immunized twice, as described in the Methods section. Four days after the second immunization, Matrigel plugs were isolated, pooled for each research subgroup, and infiltrating immune cells were analyzed by flow cytometry, as described in the Methods section. Proinflammatory cytokine levels at the vaccination site were analyzed using a Cytometric Bead Array CBA mouse inflammation kit (BD Biosciences) (**A**). Immunization with SF/H6 or miPSC/H6 increased the percentage of monocytes infiltrating the Matrigel in the vaccination site (**B**), and decreased the percentage of MDSCs. Representative plots from two separate experiments with 6 mice in each group (B). Immunization with SF/H6 or miPSC/H6 induced local production of INFγ (**C**) and IL-12p70 (**D**); Results of three experiments (6 mice in each group) (* *p* < 0.05).

**Figure 3:**
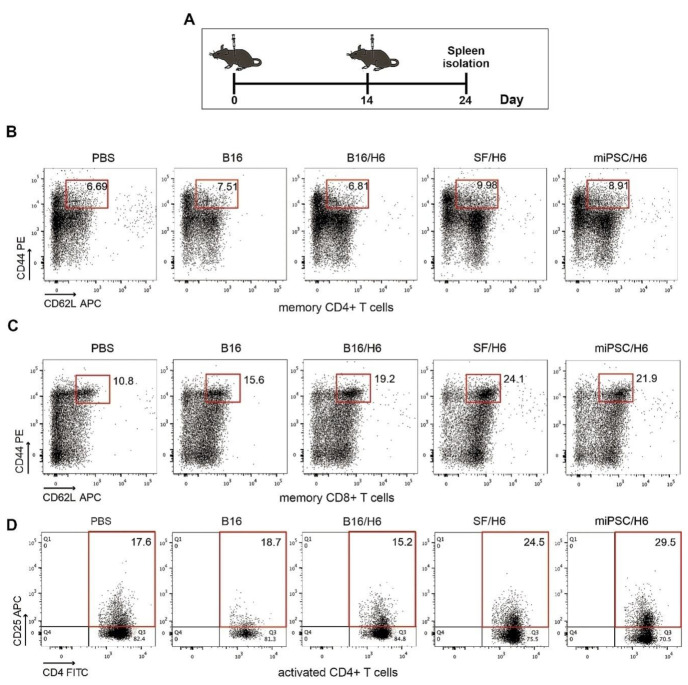
Analysis of immune cells isolated from spleens. Mice were immunized twice, as described in the Methods section. Ten days after the second immunization, spleens were collected and pooled for each research subgroup, as described in the Methods section. Cytometric analysis of isolated immune cells, T helper cytokine release assays and proliferation assays were performed (**A**). Immunization with SF/H6 or miPSC/H6 increased T cell activation (**D**) and the percentage of memory T cells in the spleen (**B, C**). Representative plots from two separate experiments with 5 mice in each group (**B**–**D**).

There was an increased percentage of inflammatory monocytes (Ly6C^+^/F4/80low cells in the CD45^+^ population) infiltrating among vaccine cells in SF/H6- and miPSC/H6-immunized mice, compared to B16/H6-immunized and control mice (Figure 2B). In contrast, there was adecreased percentage of MDSCs (Gr-1^+^/CD11b^+^ cells in the CD45^+^ population) in the same experimental groups (Figure 2C). At the site of vaccine administration, there was an increase, but no statistically significant difference, in INFγ production in immunized mice, compared to control mice (Figure 2C). There was also a significant increase in IL-12p70 production in mice immunized with SF/H6 (3-4-fold) and a statistically non-significant increase in IL-12p70 production in mice immunized with miPSC/H6, compared with control mice (Figure 2D). In spleens collected 10 days following priming, an increase in activated CD4+ T cells (CD4^+^ T cells/CD25^+^ T cells in the CD3^+^ population) was observed in SF/H6^-^ and miPSC/H6-immunized mice, compared with B16/H6-immunized and control mice (Figure 3D). The highest CD4^+^ T cell activation was observed in the miPSC/H6-immunized animals. In the same groups of mice, an increased percentage of memory CD4^+^ T cells (CD62L/CD44-expressing cells in the CD3^+^CD4^+^ population) wasobserved (Figure 3B). Compared to the control mice, the SF/H6- and miPSC/H6-immunized mice showed an increased percentage of memory CD8^+^ T cells (CD62L/CD44-expressing cells in the CD3^+^CD8^+^ population). In mice immunized with B16/H6, the percentage of memory CD8+ T cells was between that of the control and stem cell-vaccinated groups (Figure 3C).

### 3.4. Antigen-Specific CD4+ T Cell Responses

**Figure 4:**
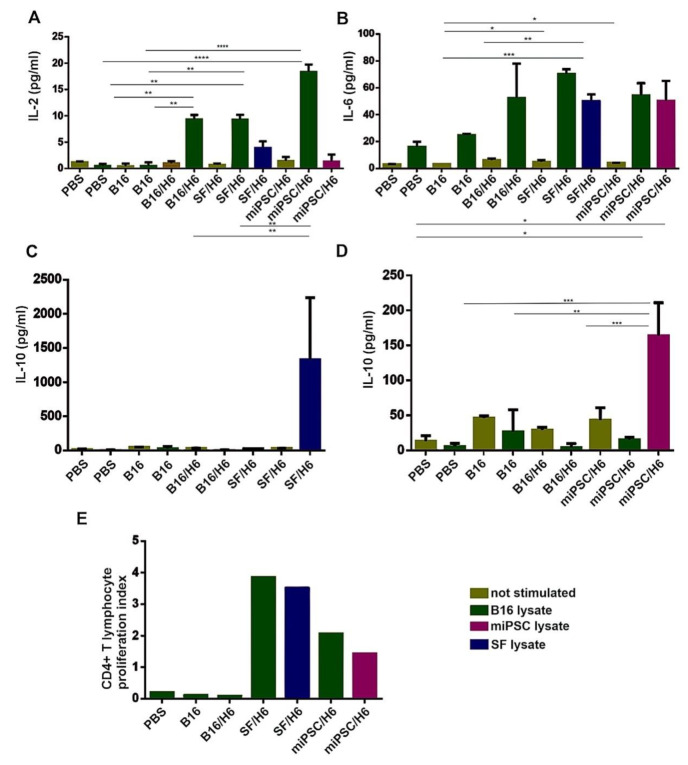
Analysis of the proliferation and cytokine production of immune cells isolated from immunized mouse spleens. Splenocytes isolated from immunized mice 10 days after priming were pooled for each research subgroup and restimulated in vitro, with lysates of B16F10 cells, SF cells or miPSCs as described in the Methods section. The concentrations of cytokines were determined using a Mouse Th1/Th2/Th17 Cytokine C kit (BD Biosciences). Immunization with SF/H6 or miPSC/H6 increased TH1/TH2 cytokine production by CD4^+^ T lymphocytes derived from spleens (**A**–**D**). Results from three experiments with 5 mice in each group, (* *p* < 0.05, ** *p* < 0.01, *** *p* < 0.001, **** *p* < 0.0001). Proliferation was evaluated using a 3H-thymidine incorporation assay. (**E**). The 3H-thymidine incorporation assay revealed increased proliferation in CD4^+^ lymphocytes derived from mice immunized with SF/H6 or miPSC/H6, compared to those derived from mice immunized with control vaccines (the result of one experiment).

In spleens, antigen specific CD4^+^ T lymphocytes were found, and these cells secreted cytokines upon re-stimulation in vitro with B16, SF or miPSC lysates. Upon re-stimulation with B16 cell lysates, an approximate 20-fold increase in IL-2 production was demonstrated in the splenocytes from mice immunized with miPSC/H6, and 10-fold increases were demonstrated with the splenocytes from SF/H6- and B16/H6-immunized mice, relative to those from PBS control mice (Figure 4A). However, in the miPSC/H6 group, no IL-2 secretion was observed upon re-stimulation with an miPSC lysate. In the miPSC/H6-immunized group, miPSC lysate re-stimulation caused a 20-fold increase in IL-10 production, compared with the B16/H6-immunized and PBS groups (Figure 4D). The increased production of IL-10 was also observed in the mice immunized with SF/H6, when splenocytes were restimulated with SF lysates (Figure 4C). No IL-10 was secreted upon restimulation in the B16/H6 group (Figure 4C,D). Increased IL-6 production was observed in the splenocytes from animals immunized with B16/H6 (2–3-fold), SF/H6 (3–4-fold) or miPSC/H6 (2–3-fold), after restimulation with B16, SF or miPSC lysates (Figure 4B). Increased proliferation of CD4^+^ T helper lymphocytes isolated from the spleens of immunized mice was found in response to restimulation with B16, SF or miPSC lysates. In cultures of splenocytes isolated from mice immunized with SF/H6, restimulation with B16 cells or SFs produced a 20–30 times higher proliferation index than the indexes for the cultures of splenocytes from B16/H6-immunized and control mice. In miPSC/H6 vaccinated mice, after restimulation with B16 or miPSC lysates, the proliferation index was approximately 10–20 times higher than that in the control mice (Figure 4E)

### 3.5. Humoral Response in Immunized Mice

**Figure 5:**
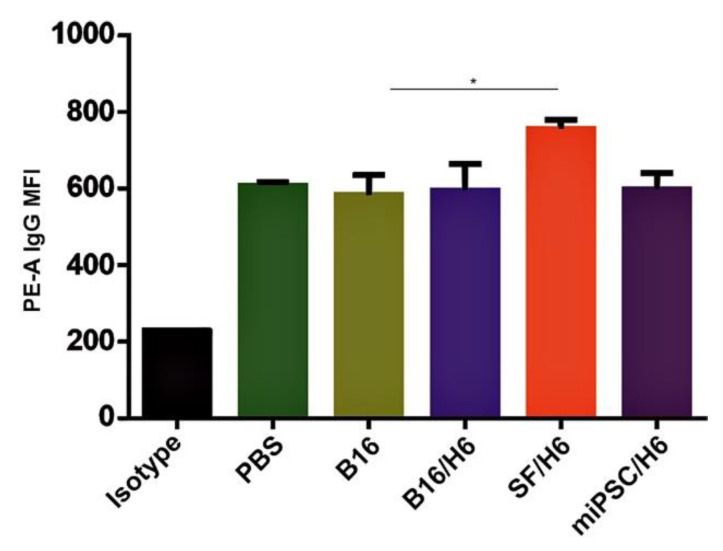
Tumor cell binding by immune serum.Serum was collected from mice 10 days after the booster vaccine administration and pooled for each research subgroup. B16F10 cells were incubated with serum containing a normalized IgG level, labeled with an anti-IgG PE antibody and analyzed by a cytometer. Immunization with SF/H6 increased the humoral response. Results of three separate experiments with 5 mice in each group (* *p* < 0.05).

Increased amounts of antibodies directed against B16F10 antigens were observed in serum samples collected from mice immunized with SF/H6, 10 days after the priming vaccination (Figure 5).

### 3.6. Effector Immune Response in the Tumor Microenvironment

**Figure 6:**
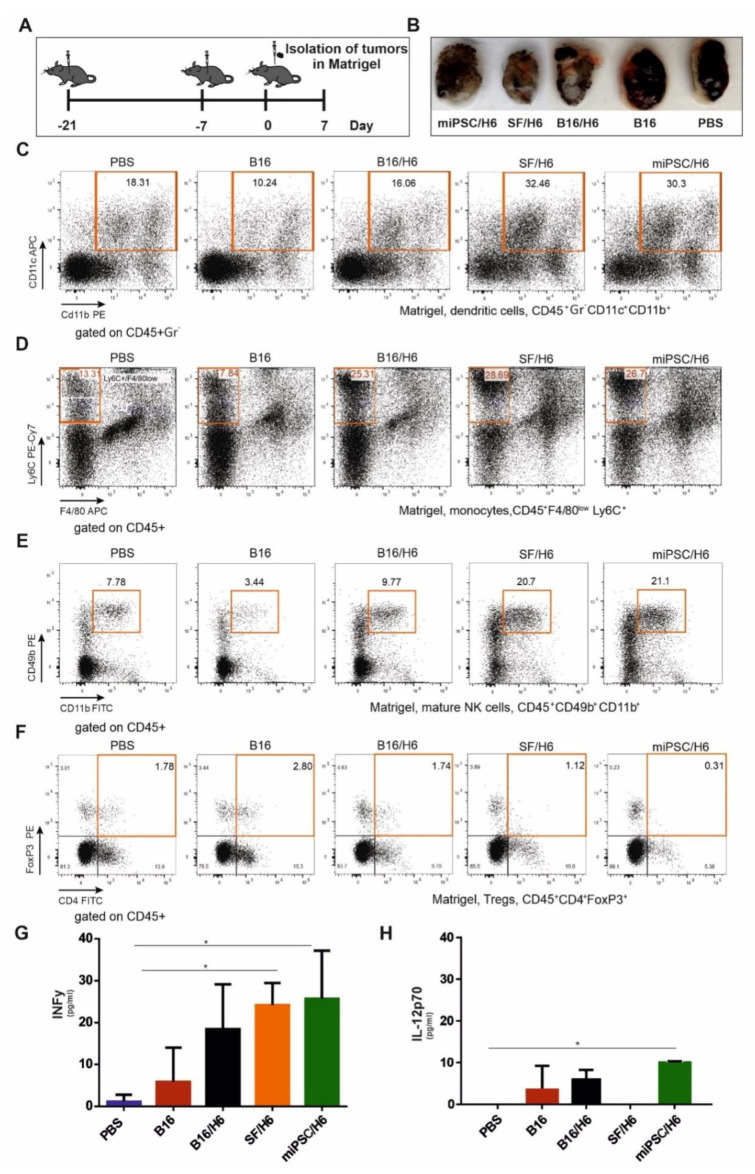
Cytometric analysis of immune cells infiltrating tumors and cytokine concentrations in the tumor site. Mice were immunized twice, as described in the Methods section (**A**). Seven days after the second immunization, mice were subcutaneously administered B16F10 tumor cells in Matrigel (A). Seven days after injection, the tumors in Matrigel were excised, pooled for each research subgroup, and infiltrating immune cells were analyzed by flow cytometry. Tumors in Matrigel excised from mice seven days after B16F10 administration (**B**). Proinflammatory cytokine levels at the tumor site were analyzed using a CBA mouse inflammation kit (BD Biosciences). Immunization with SF/H6 or miPSC/H6 increased the percentage of tumor-infiltrating dendritic cells (**C**), decreased the percentage of infiltrating monocytes (**D**), increased the percentage of infiltrating mature natural killer NK cells (**E**) and decreased the percentage of infiltrating regulatory T lymphocytes (**F**). Representative plots from two experiments with 5 mice in each group (**C**–**F**). Immunization with SF/H6 or miPSC/H6 induced the production of proinflammatory cytokines at the tumor site (**G**,**H**); results of two experiments with 5 mice in each group (* *p* < 0.05).

In contrast to mice immunized with B16/H6, SF/H6 or miPSC/H6, the control mice (PBS or B16) clearly formed tumors that were visible in the Matrigel plugs, removed seven days after B16F10 cell injection (Figure 6B).

Analysis of Matrigel-infiltrating cells showed an approximately 1.5-fold lower percentage of CD4^+^ Foxp3^+^ Treg cells in the mice immunized with SF/H6, compared to the mice immunized with B16/H6, and an approximately 2–3-fold lower percentage compared to the B16 group mice. A reduced percentage of infiltrating Tregs was observed in the miPSC/H6-immunized mice, which was approximately 5.5-fold lower than that in the B16/H6-immunized mice and 9-fold lower than that in the mice immunized with B16 cells (Figure 6F).

An increased percentage of infiltrating NK cells (CD45^+^CD49b^+^CD11b^+^) wasobserved in the SF/H6 and miPSC/H6 groups. These percentages were approximately 5 (SF/H6) or 6 (miPSC/H6) times higher than the percentage in the B16 group and approximately 2 times higher than the percentage in the B16/H6 group (Figure 6E).

In addition, 3- to 4-fold higher percentages of monocytes (Ly6^+^/F4/80low cells in the CD45^+^ population) were found in the tumors collected from mice immunized with B16/H6, SF/H6 or miPSC/H6, compared to those from mice immunized with B16 cells, and these percentages were 2-fold higher than the percentage in the PBS groups (Figure 6D). The relative amounts of infiltrating DCs (CD45^+^Gr ^–^CD11c^+^CD11b^+^) were 2-fold higher in the SF/H6 and miPSC/H6 groups than in the B16/H6 groups and 3-fold higher than in the B16 group (Figure 6C).

The highest and most significant production of INFγ in the tumor microenvironment was found in the mice immunized with SF/H6 or miPSC/H6 (Figure 6G), while the highest and most significant IL-12p70 production was observed in miPSC/H6-vaccinated mice (Figure 6H).

### 3.7. Analysis of the Clinical Effect of Immunization with the Vaccines

**Figure 7:**
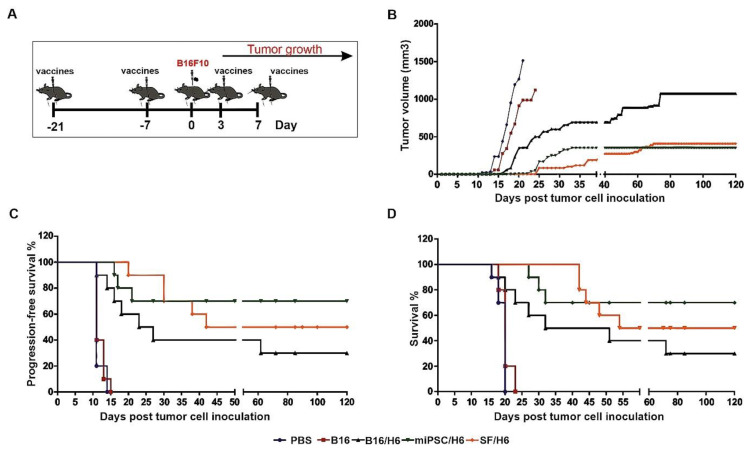
Tumor growth, disease-free survival and overall survival in immunized mice.Mice were immunized twice. Seven days after the second immunization, mice were subcutaneously administered with B16F10 tumor cells. Three and seven days later, the mice were again immunized and followed. (**A**) The SF/H6 and miPSC/H6 vaccines inhibited tumor growth (Day 20: PBS vs B16/H6, *p* < 0.0001; PBS vs. SF/H6, *p* < 0.0001; PBS vs. miPSC/H6, *p* < 0.0001; B16 vs. PBS, *p* = 0.257; B16 vs. B16/H6, *p* = 0.014; B16 vs. SF/H6, *p* < 0.0001; B16 vs. miPSC/H6, *p* < 0.0001; Day 121: B16/H6 vs. SF/H6, *p* = 0.0041; B16/H6 vs. miPSC/H6, *p* = 0.0016) (**B**) and extended disease-free survival (PBS vs. B16/H6, *p* = 0.0001; PBS vs. SF/H6, *p* < 0.0001; PBS vs. miPSC/H6, *p* < 0.0001; B16 vs. B16/H6, *p* = 0.0001, B16 vs. SF/H6, *p* < 0.0001; B16 vs. miPSC/H6, *p* < 0.0001) (**C**) and overall survival (PBS vs B16/H6, *p* = 0.001; PBS vs. SF/H6, *p* < 0.0001; PBS vs. miPSC/H6, *p* < 0.0001; B16 vs. B16/H6, *p* = 0.0016; B16 vs. SF/H6, *p* < 0.0001; B16 vs. miPSC/H6, *p* < 0.0001) (**D**). Results of two experiments with 5 mice in each group.

A significant delay in tumor take and extended DFS and OS were observed in the groups of mice immunized with the SF/H6 or miPSC/H6 vaccine. In the control groups (PBS and B16), the appearance of tumors was observed 11 days after s.c. administration of nonirradiated, wild-type B16F10 cells (Figure 7C). The tumors reached a maximum size at 20 days after cell administration (Figure 7B). On days 20 (PBS) and 22 (B16F10), the last mice were euthanized, due to tumor size exceeding 1500 mm^3^.

In mice immunized with B16/H6, the first tumors appeared 11 days after the administration of B16 cells (Figure 7C). The formation of tumors was delayed in the B16/H6-immunized mice compared to the B16F10- and PBS-immunized mice (Figure 7B). The median survival time of the mice in the B16/H6 group was 41.5 days (Figure 7D). Thirty percent of the animals survived until the end of the experiment. In the group of animals immunized with SF/H6, the first tumors appeared 20 days after B16F10 cell administration (Figure 7C). The kinetics of tumor growth were much slower in this group than in the control groups (B16 and PBS) and B16/H6 group (Figure 7B). The median survival time was 87 days, and 50% of the mice lived to the end of the experiment (Figure 7D). In mice immunized with the miPSC/H6 vaccine, the first tumors appeared 16 days after B16F10 cell administration (Figure 7C). A significant delay in tumor take was observed in this group compared to the control groups and B16/H6 group (Figure 7C). The animals immunized with miPSC/H6 lived significantly longer than the control mice and mice immunized with B16/H6. Seventy percent of the mice survived until the end of the experiment (Figure 7D).

In the final experiment, mouse tumor take and survival were compared between mice vaccinated with irradiated miPSCs (without any type of adjuvant) and those treated with PBS. Between the groups of animals, there was no difference in tumor take and no effects on tumor growth kinetics or mouse survival (Supplementary Figure S2).

## 4. Discussion

There are four major findings of this study: (i) addition of MSCs or miPSCs to a gene-modified whole-cell melanoma vaccine significantly reduced tumor take, inhibited the kinetics of tumor growth, and extended DFS and OS compared to the same vaccine alone, with miPSC addition being more effective than MSC addition; (ii) MSC or miPSC addition increased vaccine immunogenicity and antigen presentation and decreased local immunosuppression at the site of vaccine administration; (iii) stem cell addition significantly increased the effectiveness of the effector phase, including both cellular and humoral antimelanoma responses and the inhibition of tumor microenvironment immunosuppression; and (iv) miPSCs used alone, without an adjuvant, were not immunogenic and did not induce antimelanoma immune responses in mice.

The immune response comprises two phases; the induction and effector phases. The natural induction phase of the anticancer immune response is often depressed due to the low immunogenicity of cancer cells [20]. Induction may be improved by cancer vaccination, especially with whole-cell vaccines or DCs pulsed with lysates of tumor cells, which carry a broad spectrum of tumor antigens. The discovery of immune checkpoint synapses and their immunosuppressive functions in the tumor microenvironment has changed our understanding of the mechanisms governing the anticancer effector phase [21] and provided an explanation for the failure of cancer vaccines in clinical trials [22]. Moreover, the effectiveness of anti-programmed death receptor 1 (anti-PD1) therapy in melanoma in an adjuvant setting has broadened the role of these synapses in preventing tumor formation [23]. However, the above approaches display limitations, such as limited clinical response rate and limited safety (1). One of the recognized reasons for limited or transient responses to anti-PD-1 or anti-programmed death ligand 1 (anti-PDL-1) treatment is the exhaustion of naturally generated antigen-specific T cell clones in treated patients [24]. In turn, these cells can be provided by cancer vaccination. Moreover, the target cohorts of choice for cancer vaccination are patients with resected melanoma, who are immunized in the adjuvant setting. Recent animal studies [25] and our clinical observations (not published) suggest that cancer vaccination in the adjuvant setting does not prevent the systemic dissemination of cancer cells, but rather inhibits tumor formation by driving melanoma cells into dormancy.

Cancer stem/initiating cells compose a fraction of tumor cells that are resistant to different types of therapy. Due to the fact that CSCs express antigens differently from those expressed by more differentiated cancer cells, new immunotherapies targeting these CSCs have been intensively developed in recent years (3), and vaccination with (cancer) cells displaying stem like features has become ofinterest. Recently, Zhao et al. [26] demonstrated that vaccination with CSCs (CD133^+^CD44^+^) isolated from B16F10 murine melanoma cells specifically targeted B16F10-CSCs, reducing tumor growth and extending mouse survival. They also observed increased NK cell and cytotoxic splenocyte activity against B16F10 cells, as well as a reduction in NY-ESO-1 antigen expression (high in B16F10-CSCs), in melanoma tumors formed in vivo. In another study, Dashti et al. [27] used a DC vaccine pulsed with cell lysates of CSCs (CD24+CD44+), isolated from the B16F10 cell line in a prophylactic murine model. They demonstrated a significantly extended time to tumor take and prolonged OS. In immunized mice, they found the generation of specific cytotoxic T lymphocytes. T lymphocytes isolated from the lymph nodes demonstrated increased proliferation and INFγ production upon restimulation ex vivo.

Very recently, Kooreman et al. [28] demonstrated that murine autologous fibroblast-derived miPSC-based vaccines generated anticancer immunity against various types of cancers, including melanoma, breast cancer and mesothelioma. They based their studies on the thesis that iPSCs and cancer cells share a broad spectrum of epitopes. The vaccine was composed of autologous irradiated miPSCs, admixed with the Toll-like receptor 9 (TLR9) agonist CpG oligodeoxynucleotide, which served as an immunostimulatory adjuvant. The studies clearly demonstrated that prophylactic miPSC vaccination upregulated the numbers of mature antigen presenting cells in the lymph nodes, followed by increases in the numbers of helper and cytotoxic T cells, both locally and systemically. Antibodies that reacted with tumor cells, miPSCs and normal fibroblasts were also detected. It is possible that the miPSC vaccine also targeted tumor microenvironment cells, such as cancer-associated fibroblasts (CAFs). Moreover, the transfer of whole splenocytes or purified T cells from immunized mice resulted in protection against breast tumors in naïve mice. However, miPSC vaccination was not effective in the therapeutic setting in mice with established tumors. In turn, the authors debulked formed tumors and were able to restore the immune response induced by miPSC vaccination.

For the purpose of this study, we constructed whole-cell mixed melanoma vaccines composed of B16F10 cells, modified with H6 cDNA admixed with MSC-like cells or miPSCs in a 1:2 ratio. In contrast to CSCs (CD133^+^CD44^+^) isolated from B16F10 cells [26], melanosphere-derived CSCs demonstrated the downregulation of MHC class II expression. Mixing B16H6 cells with MSCs or iPSCs resulted in enhanced priming manifested by increased infiltration of WT-Matrigel tumors by DCs with decreased infiltration by MDSCs and Tregs. In the vaccination site, as well as in the tumor microenvironment, the increased infiltration of inflammatory monocytes was observed. Pommier et al. showed that this type of cell was responsible for controlling tumor dissemination in RET mice [29]. They showed that the number of monocytes was inversely proportional to the number of suppressive Tregs. Moreover, these monocytes inhibited melanocyte proliferation in vitro, via a reactive oxygen species-dependent mechanism [29]. Furthermore, we observed significantly elevated percentages of mature NK cells at the tumor site in mice immunized with SF/H6 or miPSC/H6. NK cells are capable of killing tumor cells with the reduced expression of MHC I, particularly CSCs [30,31]. At the same time, NK cells, through the production of INFγ, increase the expression of MHC I [32] on neoplastic cells and MHC II on DCs, thereby activating CD4^+^ and CD8^+^ T lymphocytes [33]. We observed increased INFγ and IL-12p70 production at the tumor sites in SF/H6 (INFγ)- and miPSC/H6 (INFγ and IL-12p70)-immunized mice. Moreover, in the same groups of mice, we found the effective activation of CD4^+^ T in spleens, as in vitro restimulation with B16, SF or miPSC lysates induced the increased proliferation of antigen-specific CD4^+^ T lymphocytes and the production of the cytokines IL-2, IL-10, and IL-6. IL-10 is a multifunctional and thus controversial cytokine. Its immunosuppressive properties are generally acknowledged; however, it has been shown that IL-10 affects the development and function of memory CD8^+^ T cells [34]. In a study, mice intravenously treated with peg-IL10 rejected implanted tumors. In addition, the increased expression of IFNγ and granzyme B, as well as a three-fold increase in tumor-infiltrating CTLs, was observed. The effects of IL-10 injection were not observed in IL-10R-/-mice [34]. IL-10 stimulates NK cells indirectly by inhibiting ROS secretion by macrophages or increasing TIA-1 expression. Moreover, IL-10 reduces MHC II expression by DCs, thereby activating NK cells and increasing their cytotoxicity [34]. Finally, the SF/H6 vaccine induced a significantly stronger antimelanoma humoral response than the B16/H6 vaccine.

## 5. Conclusions

In summary, our CSC vaccines induced a significant immune response against melanoma cells in an animal model. The effect was most potent in mice immunized with a mixed vaccine comprising iPSCs and B16/H6 cells, thus mirroring the results in human clinical trials with AGI-101H [5]. Moreover, the results of our studies indicate a novel platform for the next generation of cellular vaccine composition for the treatment of various cancer types. The platform is based on allogeneic iPSCs providing CSCs and allogeneic gene-modified cancer cell lines (cell lines delivering a broad spectrum of specific antigens for personalizing vaccination for a given cancer).

## Supplementary Materials

The following are available online at https://www.mdpi.com/2076-393X/8/2/147/s1, Figure S1. Pluripotency marker expression in miPSCs. Immunocytochemistry and qPCR were performed as described in the Methods section. iPSC expression of the surface pluripotency markers SSEA-1 (**A**), Epcam (**B**), E-cadherin (**C**), (fluorescence microscopy image (first column), phase-contrast microscopy image (second column)); expression of the transcription factor Nanog (**D**) (fluorescence microscopy image, nuclei were stained with DAPI); and alkaline phosphatase activity (**E**) (phase-contrast microscopy image). Quantitative PCR was used to assess Ssea-1 and Oct 3/4 expression in the miPSC. The results were normalized to the Gapdh expression level and are presented as the fold change relative to the expression in embryonic stem cells (** *p* < 0.01). Figure S2. Tumor growth, disease-free survival and overall survival in mice immunized with miPSCs only. Immunization with irradiated miPSCs did not affect tumor growth (**A**), progression-free survival (**B**) or overall survival (**C**). Results of one experiment with five mice in each research group.
