# Supplementary material for: Novel Genetic Melanoma Vaccines Based on Induced Pluripotent Stem Cells or Melanosphere-Derived Stem-Like Cells Display High Efficacy in a murine Tumor Rejection Model
Source: Vaccines (Basel). 2020 Mar 26;8(2):147. doi: 10.3390/vaccines8020147 (PMC7348754; doi:10.3390/vaccines8020147)
Supplement: Supplementary file 1 [file vaccines-08-00147-s001.pdf]

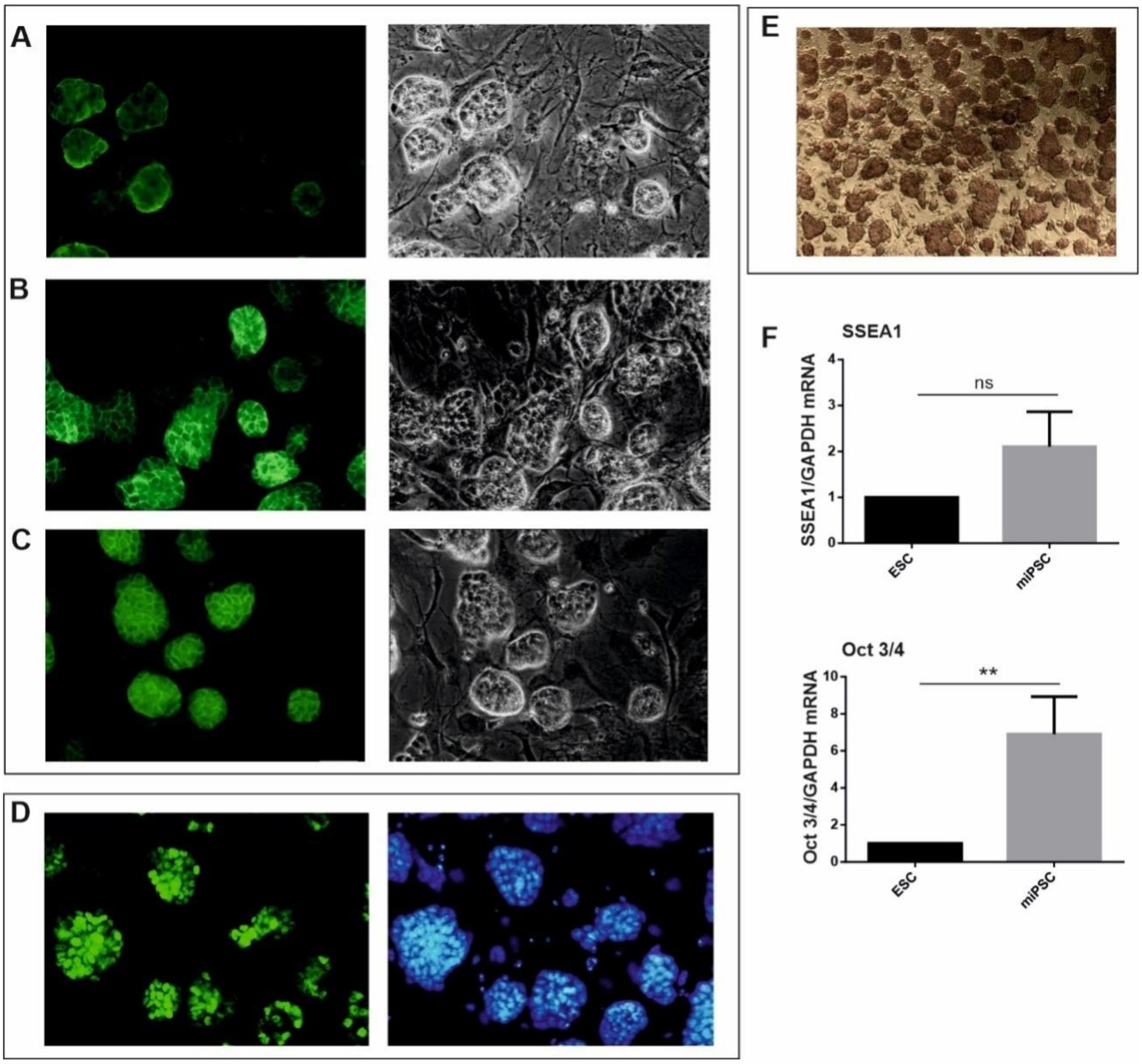

Figure S1. Pluripotency marker expression in miPSCs. Immunocytochemistry and qPCR were performed as described in the Methods section. iPSC expression of the surface pluripotency markers SSEA-1 (**A**), Epcam (**B**), E-cadherin (**C**), (fluorescence microscopy image (first column), phase-contrast microscopy image (second column)); expression of the transcription factor Nanog (**D**) (fluorescence microscopy image, nuclei were stained with DAPI); and alkaline phosphatase activity (**E**) (phase-contrast microscopy image). Quantitative PCR was used to assess Ssea-1 and Oct 3/4 expression in the miPSC. The results were normalized to the Gapdh expression level and are presented as the fold change relative to the expression in embryonic stem cells (** $p < 0.01$).

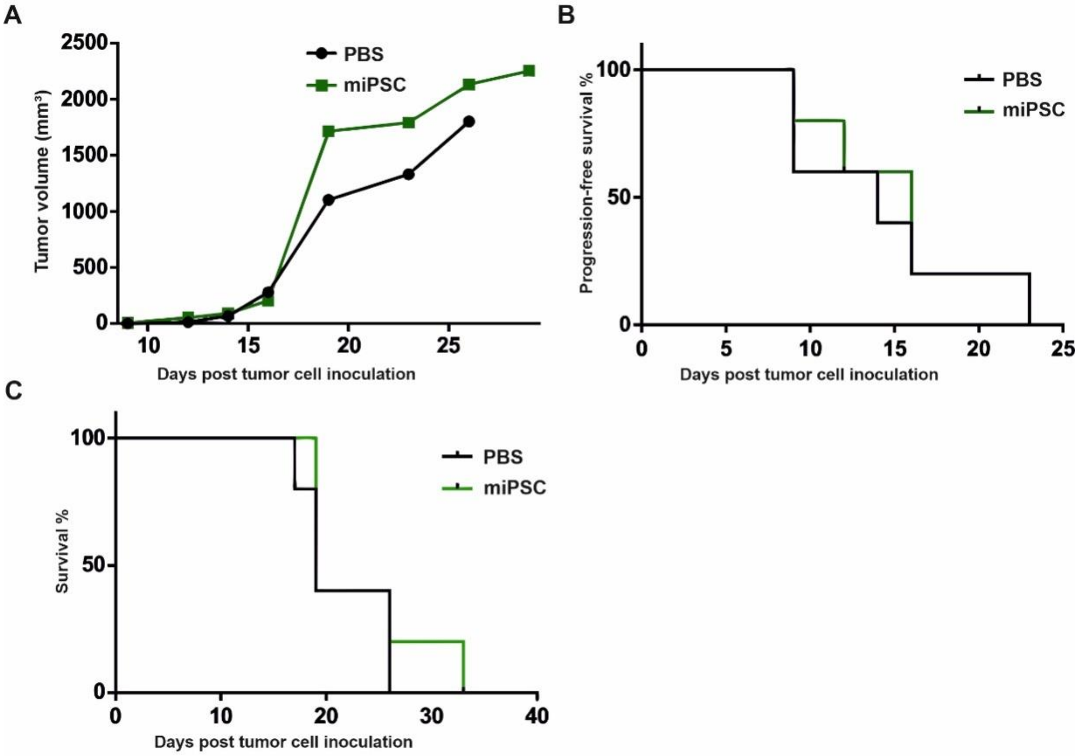

Figure S2. Tumor growth, disease-free survival and overall survival in mice immunized with miPSCs only. Immunization with irradiated miPSCs did not affect tumor growth (**A**), progression-free survival (**B**) or overall survival (**C**). Results of one experiment with five mice in each research group.
